# Neural sensitivity following stress predicts anhedonia symptoms: a 2-year multi-wave, longitudinal study

**DOI:** 10.1038/s41398-024-02818-x

**Published:** 2024-02-22

**Authors:** Jaclyn S. Kirshenbaum, David Pagliaccio, Diego A. Pizzagalli, Randy P. Auerbach

**Affiliations:** 1 Department of Psychiatry, Columbia University, New York, NY USA; 2 Division of Child and Adolescent Psychiatry, New York State Psychiatric Institute, New York, NY USA; 3 Center for Depression, Anxiety and Stress Research, McLean Hospital, Belmont, MA USA; 4 Department of Psychiatry, Harvard Medical School, Boston, MA USA; 5 Division of Clinical Developmental Neuroscience, Sackler Institute, New York, NY USA

**Keywords:** Predictive markers, Neuroscience

## Abstract

Animal models of depression show that acute stress negatively impacts functioning in neural regions sensitive to reward and punishment, often manifesting as anhedonic behaviors. However, few human studies have probed stress-induced neural activation changes in relation to anhedonia, which is critical for clarifying risk for affective disorders. Participants (*N* = 85, 12–14 years-old, 53 female), oversampled for risk of depression, were administered clinical assessments and completed an fMRI guessing task during a baseline (no-stress) period to probe neural response to receipt of rewards and losses. After the initial task run of the fMRI guessing task, participants received an acute stressor and then, were re-administered the guessing task. Including baseline, participants provided up to 10 self-report assessments of life stress and symptoms over a 2 year period. Linear mixed-effects models estimated whether change in neural activation (post- vs. pre-acute stressor) moderated the longitudinal associations between life stress and symptoms. Primary analyses indicated that adolescents with stress-related reductions in right ventral striatum response to rewards exhibited stronger longitudinal associations between life stress and anhedonia severity (*β* = −0.06, 95%CI[−0.11, −0.02], *p* = 0.008, *p*_*FDR*_ = 0.048). Secondary analyses showed that longitudinal positive associations between life stress and depression severity were moderated by stress-related increases in dorsal striatum response to rewards (left caudate *β* = 0.11, 95%CI[0.07,0.17], *p* < 0.001, *p*_FDR_ = 0.002; right caudate *β* = 0.07, 95%CI[0.02,0.12], *p* = 0.002, *p*_FDR_ = 0.003; left putamen *β* = 0.09, 95%CI[0.04, 0.14], *p* < 0.001, *p*_*FDR*_ = 0.002; right putamen *β* = 0.08, 95%CI[0.03, 0.12], *p* < 0.001, *p*_FDR_ = 0.002). Additionally, longitudinal positive associations among life stress and anxiety severity were moderated by stress-related reductions in dorsal anterior cingulate cortex (*β* = −0.07, 95%CI[−0.12,.02], *p* = 0.008, *p*_FDR_ = 0.012) and right anterior insula (*β* = −0.07, 95%CI[−0.12,−0.02], *p* = 0.002, *p*_*FDR*_ = 0.006) response to loss. All results held when adjusting for comorbid symptoms. Results show convergence with animal models, highlighting mechanisms that may facilitate stress-induced anhedonia as well as a separable pathway for the emergence of depressive and anxiety symptoms.

## Introduction

Anhedonia is characterized by a reduced ability to experience pleasure and is a cardinal symptom of major depressive disorder. During adolescence, anhedonia increases in prevalence [1], and it also coincides with increases in sensitivity to reward [2, 3] as well as exposure to stress (e.g., peer conflict, school-related problems) [4]. Animal research has consistently shown that acute stress impacts neural circuitry, often manifesting in anhedonic behaviors [5–8]. When stress occurs, the hypothalamic-pituitary-adrenal (HPA) axis secretes glucocorticoids (i.e., cortisol), which affects reward-related dopaminergic pathways [9, 10]. Dopamine is released from the ventral tegmental area (VTA) and projects to the ventral striatum, (e.g., the nucleus accumbens; NAcc) and medial prefrontal cortex, which then feeds back to the dorsal striatum (caudate, putamen) [11]. Prolonged stress can reduce dopamine availability [10, 12], and over time, reduces motivation, incentive-based learning, and social interactions [9, 13, 14]. Building on extensive animal research demonstrating that stress negatively affects neural pathways [8], the current study aimed to investigate whether neural sensitivity following acute stress exacerbated the longitudinal association between life stress and anhedonia among adolescents.

Human research shows that stressful life events impact reward functioning, which can contribute to anhedonia [15]. Research in adolescents has primarily focused on early life adversity, which associates with blunted reward-related striatal activation [16–19] and increases insula activation following reward [20]. However, insula findings may vary depending on the type of adversity [21]. The effects of acute stress on reward activation among adolescents are less clear. In adults, acute stress reduces reward-related activation in the dorsal striatum [22, 23], orbitofrontal cortex [23], and increases medial prefrontal cortex activation [24]. Additionally, we previously observed reduced ventral striatal, dorsal anterior cingulate cortex (dACC), and anterior insula activation during reward processing post-stress [25]. Overall, there is evidence that acute stress impacts cortico-striatal activation during reward processing, but further research is needed to test how this contributes to the emergence of psychiatric symptoms during a peak adolescent period of risk.

Stress also impacts neural pathways that contribute to depressive and anxiety symptoms. In depression, research consistently shows striatal blunting to rewarding stimuli, but results are less consistent regarding loss or punishment [26–30]. In the context of stress, early life stress in humans relates to blunted striatal activation to reward [19], a pattern of activity that also associates with depression severity [31]. Moreover, blunted striatal reward activation is an important potential mediator of the association between early life stress and depressive symptoms [32]. Although there are general decreases in both ventral and dorsal striatal activation in depression, there are differences between these regions. Specifically, the ventral striatum has being more consistently linked to reward valuation, and the dorsal striatum has related to goal-oriented behavior [33], including reward magnitude-tracking [34]. Moreover, although anhedonia is a symptom of depression, depression severity and anhedonia are not always associated with the same striatal regions [34–36]. Additionally, activation in the anterior insula [37] and dACC [38] is typically increased in depression, implicating possible roles in pain, salience monitoring, and tracking of loss magnitude [39–41]. Interestingly, we also have found that increased anterior insula and dACC activation in response to social rejection intensifies the longitudinal association between peer stress and depression severity [42].

In contrast, anxiety has been linked to increased striatal activity during reward processing [43–46]. However, during acute stress, research shows increased ventral but decreased dorsal striatum activation in relation to anxiety [47]. Increased insula and dACC activation in response to rejection or loss have been associated with anxiety [48, 49] and uncertainty [50]. Yet, findings are mixed regarding dACC responses to rejection in relation to early life stress [51, 52]. Together, reward-related striatal responses tend to be decreased in depression and increased in anxiety; though, this may vary between the ventral and dorsal striatum in anxiety. Moreover, both depression and anxiety tend to increase dACC and insula activation. However, whether the effects of acute stress on reward processing in these regions impact the association between psychiatric symptom and stress severity remains unclear.

The aim of the current study was to test whether neural sensitivity in reward-related regions following stress impact the longitudinal association between psychiatric symptom and stress severity (i.e., to determine whether there is evidence of an interactive effect between stress-induced brain activation during reward processing and life stress prospectively relating to psychiatric symptom severity). This study oversampled adolescents at risk of depression (by virtue of a maternal history of depression) to increase variability of psychiatric and stress symptoms over time. At the baseline assessment, participants completed a fMRI reward processing task both prior to and following an acute social stressor. Additionally, life stress exposure and psychiatric symptoms were assessed at regular intervals over a 2-year period. Our primary hypothesis was that an acute stress-related decrease in striatal response to monetary rewards would strengthen the longitudinal association between life stress and anhedonia. Our secondary hypotheses parsed associations with depression and anxiety severity. We expected that an acute stress-related decrease in striatal response to monetary rewards as well as increased dACC and anterior insula response to monetary loss would strengthen the longitudinal association between life stress and depression severity. Moreover, acute stress-related increase in striatal response to monetary rewards as well as increased dACC and anterior insula response to loss would strengthen the longitudinal association between life stress and anxiety symptom severity.

## Methods

### Participants and procedure

Adolescents (*N* = 149, ages 12–14 years-old) and their birth mothers were enrolled from the Boston metro area [25, 42]. Recruiting 12–14 year-olds at the start of the study was important to capture emergence of psychopathology over the two-year follow-up period, particularly given the median age of onset for most psychopathology is 14 years-old [53]. Additionally, the limited age range helped to constrain variation in pubertal status at the time of scan, which was important to minimize neuroendocrine differences. Adolescents were enrolled as either high-risk for depression if their mothers had experienced at least one MDD episode or low-risk for depression if their mothers had no lifetime history of MDD. Inclusion criteria for adolescent participants included right-handedness and fluency in English. Adolescent participants were excluded at baseline if they endorsed any lifetime psychiatric disorder, current psychotropic medication, neurological illnesses, or MRI contraindication.

The Partners Institutional Review Board approved study procedures. Adolescents assented and legal guardians provided written consent. During the baseline period, participants were administered clinical interviews and self-report assessments. Then, 1–2 weeks later, before the two-year follow-up data collection period, fMRI data were acquired. In the scanner, participants completed one run of the Guessing Task (no-stress condition), were exposed to an acute social stressor, and then completed a second run of the Guessing Task (stress condition). Including baseline assessments, participants completed up to 10 self-reported stress and symptom assessments 1 month, 3 months, 6 months, 9 months, 12 months, 15 months, 18 months, 21 months, and 24 months after baseline.

Following the initial clinical assessment, participants were excluded: (a) based on child and parent diagnoses (*n* = 20), (b) if they did not complete baseline clinical measures (*n* = 2), and (c) if they did not complete the MRI scan (*n* = 5). Additionally, participants were excluded if they did not finish both runs of the Guessing Task (*n* = 9), one or both scan runs contained excessive head movement (i.e., >30% of volumes with FD > 0.3 mm; *n* = 17), or if they did not complete at least two follow-up assessments (*n* = 11). The final sample included 85 participants (high-risk, *n* = 21).

### Clinical assessment

#### Clinical interviews

Clinical interviews were administered to assess lifetime psychiatric disorders for mothers (Structured Clinical Interview for DSM-IV-TR Disorders [SCID]) [54] and adolescents (Kiddie-Schedule for Affective Disorders and Schizophrenia [K-SADS]) [55]. Trained BA and MA level research assistants completed the interviews. All interviewers completed 50+ hours of training, which included didactics, mock interviews, and direct observation. Ten interview audiotapes were randomly selected (split between groups) to confirm inter-rater reliability (SCID *k* = 0.92; K-SADS *k* = 1.00).

#### Adolescent self-report measures

Participants rated their developmental stage using the Tanner Staging Questionnaire [56], which measures developmental status on a scale from 1 (no pubertal development) to 5 (adult level of pubertal development). Additionally, participants completed self-reported questionnaires of symptoms and stress. The Snaith-Hamilton Pleasure Scale (SHAPS) [57] is a 14-item questionnaire designed to measure hedonic capacity, and has been validated in adolescents [58]. Items were reverse scored, and thus, higher total scores, ranging from 14–56, reflected greater anhedonia severity (Cronbach’s ${\rm{\alpha }}$ = 0.85–0.92). The Mood and Feelings Questionnaire (MFQ) [59] is a 33-item questionnaire (score range = 0–66) assessing depression symptom severity of children and adolescents in the past 2 weeks. Higher scores indicate greater depression severity (Cronbach’s ${\rm{\alpha }}$ = 0.86–0.93). The Multidimensional Anxiety Scale for Children (MASC) [60] is a 39-item questionnaire (score range = 0–117) that measures recent anxiety symptom severity in children and adolescents. Higher scores indicate greater anxiety severity (Cronbach’s ${\rm{\alpha }}$ = 0.83–0.91). The Adolescent Life Events Questionnaire (ALEQ) [61] is a 57-item questionnaire (score range = 0–228) that measures past-month stress across family, peer, romantic, and academic domains. Higher scores indicate greater stress severity.

### fMRI task

The Guessing Task [62] probes brain activation following the receipt of monetary reward and loss feedback. For each trial, there was a jittered inter-trial interval, which presented a fixation cross for 1300–9100 ms. Then, participants viewed two identical doors side-by-side and were instructed to select the door they thought contained a reward as quickly as possible by pressing the left or right button on the button box, respectively. Participants were instructed that for each trial, there was an option to win $ 0.50 behind one of the doors or lose $ 0.25 behind the other door. The doors were presented for up to 3900 ms, after which the doors disappeared. After a brief fixation cue, feedback was displayed for 1300 ms: either a green ‘↑’ indicating a correct guess (Reward Feedback) or a red ‘↓’ reflecting an incorrect guess (Loss Feedback). Participants completed 48 trials. Unbeknownst to participants, the outcome was fixed, as participants received equivalent win and loss feedback in pseudorandom order. This task was administered twice, which occurred both before (no-stress condition) and after (post-stress condition) the acute stressor.

### Acute stress manipulation

Prior to entering the scanner, participants rated their positive (i.e., happy, joyful) and negative (i.e., upset, discouraged) affect on a visual analog scale from 0 (not very true of me) to 100 (very true of me). After the first run of the Guessing Task, participants completed the Chatroom Task [44, 63], which is designed to probe neural processes related to social feedback. Briefly, participants completed an online profile, rated profiles of age- and gender-matched peers, and then, were informed that peers from collaborating institutions would review their profiles and indicate whether they were interested (i.e., peer acceptance) or not interested (i.e., peer rejection) in chatting online with them [42]. The Chatroom MRI Task was then used as an acute stressor. Specifically, following the completion of the task, a screen was displayed with the following feedback, “Individual Performance: Peer Acceptance: 38%, Peer Rejection: 62%; Average Participant Performance: Peer Acceptance: 64%, Peer Rejection: 36%.” Study staff explained the feedback with the following, “*Based on the breakdown from today, it seems like you’re accepted by fewer teens compared to other teens completing the task. Additionally, you are being rejected more than other teens that have completed the selection process.*” The second part of the stressor included the rationale for re-doing The Guessing Task. For this part, study staff read the following statement to participants, “*Unfortunately, your performance in the Guessing Task was below average. Remember, you earned only $6 out of a possible $24. For the data to be usable, a participant needs to earn more than $* *12.* *Thus, we’re going to need to redo this task. Please try to focus*.” After study staff read these statements aloud, participants rated how they felt on the same visual analog scale that was administered prior to entering the scanner, which was followed by completing the second run of The Guessing Task.

### fMRI data acquisition and preprocessing

MRI data were acquired on a Siemens Tim Trio 3 T MR scanner using a 32-channel head coil. A multi-echo magnetization prepared gradient echo (MP-RAGE) T1-weighted sagittal anatomical image was obtained (repetition time [TR] = 2200 ms; echo time [TE]1 = 1.54 ms, TE2 = 3.36 ms. TE3 = 5.18 ms, TE4 = 7 ms; flip angle = 7°; field-of-view [FOV] = 230 mm; voxel size = 1.2 mm isotropic; 144 slices). Two runs of functional data during the Guessing Task were acquired with T2*-weighted gradient echo-planar functional imaging sequences, each with 491 volumes (TR = 1300 ms; TE = 32.2 ms; echo spacing=0.69 ms; flip angle=66°; FOV = 212 mm; 72 slices; voxel size = 2 mm isotropic, multiband acceleration factor = 8). A field map was acquired for distortion correction (TR = 1000 ms; TE1 = 10 ms, TE2 = 12.46 ms; voxel size = 3.5 × 1.8 × 2.5 mm; 51 slices).

Preprocessing was performed using fMRIPrep v1.5.10 [64, 65]; (RRID:SCR_016216), which is based on *Nipype* 1.4.2 [66, 67]; (RRID:SCR_002502). See Supplementary Material for details.

### fMRI analysis

AFNI [68, 69] was used to postprocess data and perform first-level GLMs using 3dDeconvolve, separately for Pre-stress and Post-stress conditions. First, the number of voxel outliers at each volume of the timeseries using 3dToutcount were calculated. Second, the data were spatially smoothed using 3dBlurInMask with a 4 mm FWHM kernel, and a functional mask was created per participant using 3dAutomask to remove areas with signal dropout. Third, functional data were rescaled (Mean = 100, range = 0–200). Last, we constructed GLMs (3dDeconvolve), which regressed the 32 motion confounds and volumes denoted as outliers. The GLMs also included regressors for the doors trials with standard gamma HRF (GAM) or a gamma function convolved with a variable duration boxcar (dmBLOCK): (1) Loss Feedback (Loss > Baseline) and (2) Win Feedback (Win > Baseline). Baseline was defined with the inter-trial interval. We fit models with restricted maximum likelihood estimation of temporal auto-correlation structure using 3dREML.

### Motion correction

Several steps were undertaken to reduce the effects of motion artifact. Based on Fair et al. (2020), a notch filter was applied with minimum and maximum respiratory rates of 0.31 Hz and 0.43 Hz, respectively [70] to the 6-parameter head motion estimates to remove respiration-related effects. TRs exhibiting large motion (≥ 0.3 mm) between successive TRs were regressed out (i.e., denoted as an outlier) in addition to TRs where at least 5% of brain voxels were computed as timeseries outliers.

### ROI selection

Nine a priori ROIs (Supplementary Fig. 1) were selected based prior literature and on our previous work with a subset of the current sample [25]. Reward-related ROIs included the left and right nucleus accumbens, caudate, and putamen extracted from the probabilistic Harvard-Oxford subcortical atlas at a 50% threshold. Loss-related ROIs included the dACC and left and right anterior insula. Neurosynth (www.neurosynth.org; [71]) was used to obtain a reward mask (uniformity test: *p* < 0.01 FDR corrected), which included the dorsal anterior cingulate cortex (dACC) and left and right anterior insula. All ROIs were resampled to 2 mm isotropic voxels and gray-matter masked (MNI152Nlin2009cAsym at 25% probability threshold). Authors visually confirmed ROI coverage of each participant’s signal (3dAutomask) across pre- and post-stress conditions. Sensitivity analyses were conducted to exclude participants who did not exhibit full coverage of ROIs. Finally, the mean activation for all non-zero voxels within each ROI was extracted for each participant using 3dROIstats. To obtain estimates of change, post-stress activation was regressed onto pre-stress activation, which yielded standardized residuals for each region.

### Data analytic approach

Analyses were conducted in Rv4.2.2 [72]. First, anhedonia (SHAPS), depression (MFQ), and anxiety (MASC) symptoms were Winsorized if values exceeded Q3 ± 3*IQR. Second, to determine which variables to include as covariates, linear mixed-effects models were performed without brain ROIs. Models included the following variables: life stress (ALEQ), age, sex, pubertal stage (Tanner), risk group, and visit number. Separate linear effects models were conducted with anhedonia, depression, and anxiety symptoms as the dependent variables. To disaggregate the between- and within-person effects of life stress, all models included a level-2 fixed effect for sample-centered mean stress score (i.e., between-person, or time-invariant) and a level-1 person-mean-centered stress score (i.e., within-person stress, or time-varying). A random intercept of person and random slope of visit number were included, and 95% confidence intervals were bootstrapped.

Third, following determination of covariates for each psychiatric outcome, linear mixed-effects models estimated whether change in ROI activation (post- vs. pre-acute stressor) moderated the longitudinal associations between within-person life stress and symptoms. False discovery rates (FDR) corrected for six multiple tests probing reward-related regions (left and right NAcc, left and right caudate, left and right putamen) and three multiple tests probing loss-related regions (dACC, left and right anterior insula). After determining significant interaction effects, separate models were conducted to covary the other longitudinal (i.e., baseline through follow-up) psychiatric symptom data (e.g., adjusting for anxiety and depression when anhedonia is the dependent variable). Code is available at: https://github.com/jackie-schwartz/neural_sensitivity_to_stress.

## Results

### Preliminary analyses

Participant characteristics are summarized in Table 1. Differences in sociodemographic or clinical characteristics among retained and excluded participants are included in Supplementary Tables 1 and 2. Correlations among stress and psychiatric symptoms are included in Table 2. Means and standard deviations of psychiatric symptoms across follow-up assessments are presented in Supplementary Table 3. An average of eight follow-up visits were completed. For results on change in ROI activation pre- to post-stress see Supplementary Figs. 2 and 3.

**Table 1:** Participant characteristics.

Variable	Total (*N* = 85)	Low risk (*n* = 64)	High risk (*n* = 21)	Group difference
Age in years (M, SD)	12.99, 0.79	12.98 (0.81)	13 (0.77)	*t* = −0.08, *p* = 0.937
Tanner Score (M, SD)	3.04, 0.60	3.07 (0.58)	2.98 (0.65)	*t* = 0.56, *p* = 0.580
Sex: Female (%)	62	59	71	X2 = 0.533, *p* = 0.466
Ethnicity: Hispanic (%)	4	5	0	X2 = 0.11, *p* = 0.742
Race (%)				
Asian	5	6	0	X2 = 5.39, *p* = 0.068
Black	0	0	0	
Multiracial	8	5	19	
White	87	89	81	
Income (%)				
50–75k	7	6	9	X2 = 1.39, *p* = 0.708
75–100k	15	14	19	
100k+	67	67	67	
Unknown	11	13	5	
Anhedonia (M, SD)	22.01 (4.64)	21.22 (4.60)	24.43 (3.97)	*t* = −3.09, *p* = 0.004
Depression (M, SD)	7.05 (6.51)	6.47 (5.67)	8.81 (8.51)	*t* = 1.18, *p* = 0.250
Anxiety (M, SD)	35.75 (12.03)	33.58 (11.45)	42.38 (11.56)	*t* = −3.03, *p* = 0.005
Stress (M, SD)	20.04 (14.14)	20.09 (14.27)	19.86 (14.08)	*t* = 0.07, *p* = 0.947

Choices for biological sex were Male or Female.

**Table 2:** Repeated measures correlation table.

	Anhedonia (SHAPS)	Depression (MFQ)	Anxiety (MASC)
Anhedonia (SHAPS)			
Depression (MFQ)	0.23**		
Anxiety (MASC)	0.07*	0.46**	
Total Stress (ALEQ)	0.17**	0.49**	0.30**

This table presents Pearson correlation coefficients for repeated measures data. *p < 0.05, **p < 0.001.

*SHAPS* Snaith-Hamilton Pleasure Scale, *MFQ* Mood and Feelings Questionnaire, *MASC* Multidimensional Anxiety Scale for Children, *ALEQ* Adolescent Life Events Questionnaire.

With regards to the acute stress manipulation, there was a significant decrease in positive affect ratings pre- (*M* = 75.91) to post-stress (*M* = 46.82), *t*(149.95) = -9.12, *p* < 0.001 (Cohen’s *d* = 1.42), as well as a significant increase in negative affect ratings pre- (*M* = 12.27) to post-stress (*M* = 46.77), *t*(147.73) = 10.87, *p* < 0.001 (Cohen’s *d* = 1.70) (Supplementary Fig. 4), suggesting the acute stress manipulation achieved the desired effect.

In models identifying potential covariates, between-person life stress (ALEQ) was associated with anhedonia severity ($\beta$ = 0.15, 95%CI[0.00, 0.31], *p* = 0.044), depression severity ($\beta$ = 0.54, 95%CI[0.41, 0.66], *p* < 0.001), and anxiety severity ($\beta$ = 0.34, 95%CI[0.18, 0.49], *p* < 0.001). Age at baseline was negatively associated with anhedonia ($\beta$ = −0.26, 95%CI[−0.45, −0.09], *p* = 0.004) and depression severity $(\beta$ = −0.21, 95%CI[−0.35, −0.06], (*p* = 0.004); however, pubertal stage at baseline was only associated with depression severity ($\beta$=.17, 95%CI[0.00, 0.32], *p* = 0.046). Sex (females > males) was associated with anhedonia ($\beta$ = −0.43, 95%CI[−0.74, −0.11], *p* = 0.008) and anxiety severity$(\beta$ = 0.55, 95%CI[0.22, 0.88], (*p* < 0.001). Risk Group (high > low) was only associated with anhedonia severity ($\beta$ = 0.69, 95%CI[0.35, 1.03], *p* < 0.001), but not with depression or anxiety severity (ps ≥ 0.066). Visit number was not associated with any psychiatric symptoms (*ps* ≥ 0.184).

### Primary analysis: predicting anhedonia symptoms

**Fig. 1:**
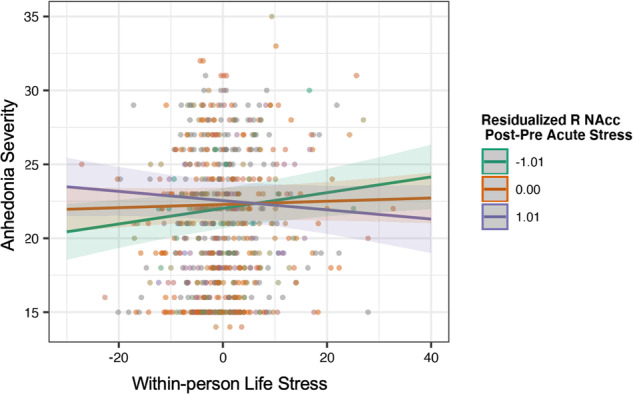
Right nucleus accumbens activation post-stress moderates the longitudinal association between life stress and anhedonia severity. Note: model adjusting for age, risk group, sex, between-person stress, and longitudinal.

An acute stress-related reduction in the right NAcc activation to win significantly moderated the association between longitudinal life stress exposure and anhedonia severity, adjusting for age, Risk Group, sex, and between-person stress (*β* = −0.06, 95%CI[−0.11, −0.02], *p* = 0.008, *p*_*FDR*_ = 0.048). Covariates of age (*β* = −0.23, 95%CI[−0.37, −0.07]), Risk Group (high > low; *β* = 0.70, 95%CI[0.36, 1.03]), Sex (females > males; *β* = −0.42, 95%CI[−0.72, −0.11]), and between-person stress (*β* = 0.15, 95%CI[0.00, 0.30]) remained significantly associated with anhedonia severity (*p*s ≤ 0.042). Importantly, after separately covarying longitudinal depression and anxiety symptoms, the interactions remained significant (*ps* ≤ 0.01, see Fig. 1). Simple slopes analyses indicated that the association between life stress and anhedonia was reduced among those with stress-related increase in NAcc activation (*β* = 0.02, 95%CI[−0.05, 0.09], *p* = 0.590) compared to average activation (*β* = 0.09, 95%CI[0.04, 0.13], *p* < 0.05) and activation one standard deviation below the mean (*β* = 0.15, 95%CI[0.08, 0.22], *p* < 0.05). Results remained significant in sensitivity analyses excluding three participants missing up to 10 voxels of NAcc coverage (ps < 0.018, see Supplementary Table 4). There were no other significant interactions associated with follow-up anhedonia symptoms (Supplementary Tables 5, 6).

### Secondary analyses: predicting depression symptoms and anxiety symptoms

**Fig. 2:**
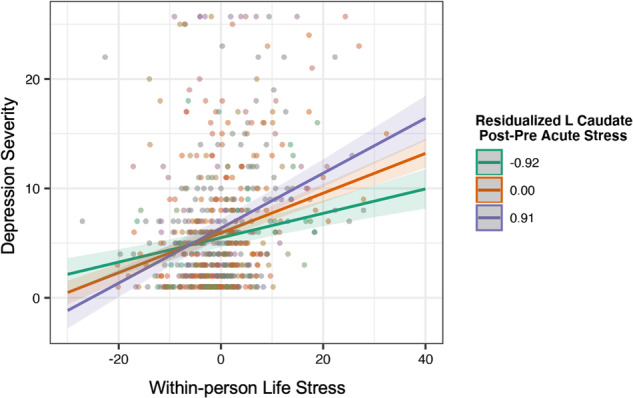
Left caudate activation post-stress moderates the longitudinal association between life stress and depression severity. Note: model adjusting for age, Tanner, between-person stress, and longitudinal anxiety severity. The R caudate, and L and R putamen as moderators show similar effects. Adjusting for longitudinal anhedonia severity reveals similar associations.

Stress-related change in left and right caudate and left and right putamen responses to monetary gains moderated the longitudinal association between stress and depression symptoms, adjusting for age, Tanner, and between-person stress (left caudate *β* = 0.11, 95%CI[0.07,0.17], *p* < 0.001, *p*_FDR_ = 0.002; right caudate *β* = 0.07, 95%CI[0.02,0.12], *p* = 0.002, *p*_FDR_ = 0.003; left putamen *β* = 0.09, 95%CI[0.04, 0.14], *p* < 0.001, *p*_*FDR*_ = 0.002; right putamen *β* = 0.08, 95%CI[0.03, 0.12], *p* < 0.001, *p*_FDR_ = 0.002). In the model testing the interaction between the left caudate and within-person stress, covariates of age (*β* = −0.21, 95%CI[−0.34, −0.06]), Tanner (*β* = 0.16, 95%CI[0.00, 0.30]), and between-person stress (*β* = 0.57, 95%CI[0.44, 0.70]) remained significantly associated with depression severity (*p*s ≤ 0.048). In the model testing the interaction between the right caudate and within-person stress, covariates of age (*β* = −0.20, 95%CI[−0.34, −0.06]) and between-person stress (*β* = 0.57, 95%CI[0.44, 0.69]) remained significantly associated with depression severity (*p*s ≤ 0.004); however, Tanner was no longer significantly associated with depression severity (*β* = 0.14, 95%CI[−0.01, 0.29], *p* = 0.076). Similarly, in the model testing the interaction between the left putamen and within-person stress, covariates of age (*β* = −0.20, 95%CI[−0.34, −0.06]), Tanner (*β* = 0.17, 95%CI[0.01, 0.31]), and between-person stress (*β* = 0.55, 95%CI[0.43, 0.68]) remained significantly associated with depression severity (*p*s < 0.040). Finally, in the model testing the interaction between the right putamen and within-person stress, covariates of age (*β* = −0.20, 95%CI[−0.34, −0.06]), Tanner (*β* = 0.16, 95%CI[0.01, 0.31]), and between-person stress (*β* = 0.56, 95%CI[0.43, 0.69]) remained significantly associated with depression severity (*p*s < 0.044). Contrary to our hypotheses, simple slopes analyses indicated that participants with *higher* stress-related increase in brain response to rewards showed the strongest associations between life stress and depression severity. The association between life stress and depression severity was strongest among those with stress-related increase in left caudate activation (*β* = 0.40, 95%CI[0.33, 0.47], *p* < 0.05) and right caudate activation (*β* = 0.35, 95%CI[0.29, 0.42], *p* < 0.05) compared to average activation (left caudate *β* = 0.30, 95%CI[0.26, 0.34], *p* < 0.05; right caudate *β* = 0.29, 95%CI[0.25, 0.33], *p* < 0.05) and activation one standard deviation below the mean (left caudate *β* = 0.19, 95%CI[0.14, 0.25], *p* < 0.05; right caudate *β* = 0.23, 95%CI[0.17, 0.28], *p* < 0.05). Similarly, the association between life stress and depression severity was strongest among those with stress-related increase in left putamen activation (*β* = 0.36, 95%CI[0.30, 0.42], *p* < 0.05) and right putamen (*β* = 0.37, 95%CI[0.31, 0.43], *p* < 0.05) compared to average activation (left putamen *β* = 0.28, 95%CI[0.24, 0.32], *p* < 0.05; right putamen *β* = 0.28, 95%CI[0.24, 0.32], *p* < 0.05) and activation one standard deviation below the mean (left putamen *β* = 0.20, 95%CI[0.13, 0.26], *p* < 0.05; right putamen *β* = 0.20, 95%CI[0.14, 0.26], *p* < 0.05). These models remained significant when removing three potential outlier observations (*ps* ≤ 0.002), one participant missing 2 voxels of putamen coverage (*ps* < 0.001, see Supplementary Table 7), as well as when adjusting for longitudinal anxiety and anhedonia symptoms (*ps* ≤ 0.002; see Fig. 2). No significant interactions of the NAcc emerged (Supplementary Table 8) or within the loss condition emerged (Supplementary Table 9).

**Fig. 3:**
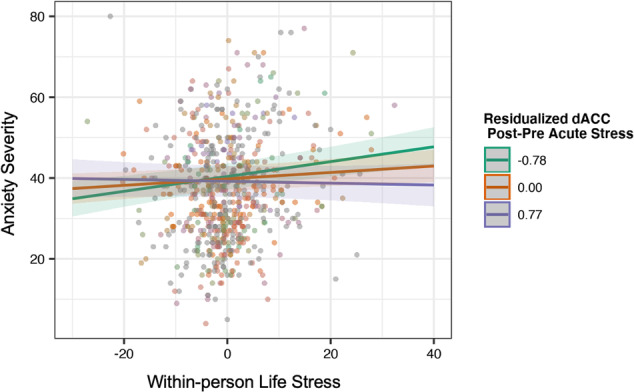
Dorsal anterior cingulate cortex activation post-stress moderates the longitudinal association between depression severity. Note: model adjusting for sex, between-person stress, and longitudinal depression severity. The R anterior insula shows similar effects. Adjusting for longitudinal anhedonia severity reveals similar associations.

Stress-related change in dACC activation (*β* = −0.07, 95%CI[−0.12,−0.02], *p* = 0.008, *p*_FDR_ = 0.012) and right anterior insula activation (*β* = −0.07, 95%CI[−0.12,−0.02], *p* = 0.002, *p*_*FDR*_ = 0.006) to loss moderated the association between follow-up stress and anxiety, adjusting for sex and between-person stress. In the model testing the interaction between the dACC and within-person stress, covariates of Sex (females > males) (*β* = 0.58, 95%CI[0.24, 0.91]) and between-person stress (*β* = 0.36, 95%CI[0.21, 0.52]) remained significantly associated with anxiety severity (*p*s < 0.001). Similarly, in the model testing the interaction between the right anterior insula and within-person stress, covariates of Sex (females > males) (*β* = 0.56, 95%CI[0.25, 0.88]) and between-person stress (*β* = 0.35, 95%CI[0.21, 0.51]) were significantly associated with anxiety severity (*p*s < 0.001). Importantly, after adjusting for longitudinal depression and anhedonia symptoms during the follow-up periods, the interactions remained significant (*ps* ≤ 0.008; Fig. 3). Contrary to our hypotheses, simple slopes analyses indicated that participants with a greater stress-related decrease in brain response to loss showed the strongest associations between life stress and anxiety severity. The association between life stress and anxiety severity was strongest among those with stress-related decrease in dACC activation (*β* = 0.20, 95%CI[0.15, 0.26], *p* < 0.05) compared to average activation (*β* = 0.15, 95%CI[0.11, 0.19], *p* < 0.05) and activation one standard deviation above the mean (*β* = 0.10, 95%CI[0.04, 0.15], *p* < 0.05). Similarly, the association between life stress and anxiety severity was strongest among those with stress-related decrease in right anterior insula activation (*β* = 0.20, 95%CI[0.15, 0.25], *p* < 0.05) compared to average activation (*β* = 0.14, 95%CI[0.10, 0.18], *p* < 0.05) and activation one standard deviation above the mean (*β* = 0.08, 95%CI[0.03, 0.14], *p* < 0.05). There also were no significant interactions between the dorsal or ventral striatum and stress within the win condition predicting follow-up anxiety severity (Supplementary Table 10) or between the left anterior insula and stress in the loss condition (Supplementary Table 11).

## Discussion

Stress strongly impacts reward functioning and, for some, alters incentive processing [14, 73, 74]. Stress exposure increases risk for psychiatric symptoms [75, 76], but the mechanisms through which stress leads to these symptoms remains unclear. Animal models have implicated dysfunction of dopaminergic system, and although challenging in humans, it is possible to indirectly probe dopaminergic neural processes during stress-related change. Accordingly, we implemented an acute stress manipulation to test whether change in neural activation to monetary rewards and losses pre-to-post stress moderated the longitudinal association between life stress exposure and psychiatric symptoms.

In line with our hypothesis, decreased activation of the ventral striatum (right NAcc) to rewards pre-to-post-stress moderated the longitudinal association between life stress and anhedonia in adolescents. Specifically, individuals with stress-related reduction in striatal response to rewards showed a stronger association between life stress and anhedonia, whereas those exhibiting stress-related increases showed a blunted association between life stress and anhedonia. Acute stress temporarily recruits dopamine to engage in adaptive learning and coping mechanisms [9, 77]. Thus, a reduced response to rewards following acute stress may indicate greater sensitivity to the effects of future life stressors and a more anhedonic phenotype (i.e., diminished pleasure or motivation). Although this association was identified in the right NAcc, we did not observe a significant effect in the left NAcc. Consistent with the lateralization of our ventral striatum findings, Webb and colleagues [78] found that greater pre-treatment right, but not left, striatal response to wins predicted greater improvement in anhedonia post-treatment in adolescents. However, Eckstrand et al. (2019) reported the left, but not right, activation of the ventral striatum to rewards associated with improved longitudinal severity in young adults [79], and there are also reports of bilateral activation in the ventral striatum in relation to anhedonia [30]. It is possible that developmental changes in lateralization of the ventral striatum in relation to stress and anhedonia occur throughout adolescence. Contrary to our hypotheses and to previous research [36, 80–82], we did not find that the dorsal striatum moderated the association between stress and anhedonia. Although the dorsal and ventral striatum have shown blunted reactivity to rewards, these regions are functionally distinct in their reward processing roles. Thus, whereas the ventral striatum is involved mainly in reward valuation [33, 39], the dorsal striatum is often involved in response inhibition and action-dependent decision making [23, 83]. Further research comparing ventral and dorsal activation probing different aspects of reward processing (e.g., reward learning) may clarify specificity of the striatum as it relates to stress and risk for the unfolding of anhedonia symptoms.

The striatum is part of a larger cortico-striatal circuit that includes the insula and anterior cingulate cortex [84], which are regions typically recruited during stressful situations to help guide attention and shift goal-directed behaviors [85]. In our study, we did not find that the change in activation to loss in the dACC and anterior insula following acute stress moderated the association between life stress and anhedonia. Given evidence of dACC and anterior insula activation relating to anhedonia particularly in the context of uncertain reward cues [86] and estimation of effort associated with rewards and costs [87], it may be that change in activation to loss in these regions following stress relates more to apprehension dimensions of anhedonia [88], rather than general anhedonia.

Our secondary aim was to test whether neural sensitivity to stress moderates the association between life stress and depression and anxiety symptom severity. Contrary to our hypotheses, increased dorsal striatal (putamen and caudate) activation to rewards post-stress moderated the longitudinal association between life stress and depression severity. Reductions in dorsal striatal activation during reward processing have been implicated in depression [34, 80], and dorsal striatal activity to rewards typically decreases following acute stress [23]. Twenty-five percent of our sample had mothers with a history of depression, which commonly co-occurs with other disorders (e.g., addiction, anxiety, eating disorders) [89, 90]. It is possible that risk for these comorbidities may be reflected in striatal activation patterns observed. Additionally, although both are parts of the reward circuitry, the ventral and dorsal striatum normatively follow different spatial trajectories over the course of adolescent development [91]. Our small age range may be capturing a unique period of striatal differences in response to reward and stress. Given our sample was psychiatrically healthy and young at the time of scan, it is possible the dorsal striatum played a compensatory role in response to acute stress, which may serve as a marker of longer-term stress-depression associations. Studies over longer periods of time are necessary to test differences in the trajectories of ventral and dorsal striatal responses to reward in the context of acute stress.

Contrary to our hypothesis, reduced dACC and right anterior insula activation to loss post-stress moderated the longitudinal association between life stress and anxiety severity. The dACC and anterior insula are key regions of the salience network, which are connected to subcortical regions of the striatum, and implicated in response to uncertainty and salient environmental cues [92]. Our findings conflict with previous research linking heightened activation of salience network regions with anxiety [93]. Although, one study found that stress-induced activation of the dorsal ACC and anterior insula was not related to anxiety in adolescents [47], other studies have found activation of the ACC and insula is negatively associated with anxiety in youth [94, 95], possibly reflecting inflexibility responding to change or error, particularly from childhood to adolescence. Given the dACC and insula are regions that also help to engage cognitive control [96], it is possible that a stress-induced decrease in dACC and anterior insula activation to loss in our study reflects a difficulty to flexibly respond to future life stress.

Although our study has important strengths (e.g., within-scanner stress manipulation, longitudinal psychiatric and stress data), it also has some limitations. First, although adolescents repeatedly reported on their psychiatric symptoms and life stress for up to 10 times over the course of two years, which allowed us to detect within-person effects, our sample size was relatively small to detect between-person individual differences. Second, the reward paradigm was repeated within a single session to gauge neural responses to acute stress, but it was not repeated during the follow-up. Future research may explore whether developmental differences in neural sensitivity to stress impact the association between psychiatric symptoms and life stress. Third, although counterbalancing would help ensure that the changes in affect pre-to-post-stress were due to stress rather than fatigue, the design of the task, which relies on repetition to probe within-person change, does not make counterbalancing feasible. Future research could add a comparison group, where the manipulation does not include the stress component. Fourth, although our sample included a portion of adolescents who were at high risk for depression given their maternal history, adolescents were recruited to be psychiatrically healthy at the time of the scan. Unexpected findings may be due to this sample composition. Results may differ in a more clinically acute sample of adolescents. Although underpowered in the current study, future research may examine gender differences in relation to stress and depression, as this age range coincides with when depression rises more rapidly for girls than for boys. Additionally, our sample were mostly White with a high socioeconomic status limiting the generalizability of our results to other populations, which can be explored further in future work.

Our study expanded on animal studies examining the effects of acute stress by investigating stress-related change in neural activation within an fMRI paradigm in relation to several assessments of anhedonia and life stress symptoms. Similar to other research [42], our findings can be interpreted in the context of diathesis-stress models, as stress-related changes in the ventral striatum may serve as a vulnerability marker that increases risk for heightened anhedonia severity when life stress occurs. Stress-related changes in the striatum, dACC, and anterior insula may also differentially moderate the association between life stress and other related psychiatric symptoms, such as depression and anxiety. As adolescents experience new life stressors and increases in internalizing problems, our findings may shed light on potential neurobiological mechanisms that link the long-established associations between life stress and internalizing symptoms. Additionally, given that change in neural activation prospectively associated with stress and psychiatric symptoms, neural activation during reward processing may be a modifiable process that can have long-term effects.

### Supplementary information

Supplemental Material

## Data Availability

Data will be made available upon request.
