# Supplementary material for: Neural sensitivity following stress predicts anhedonia symptoms: a 2-year multi-wave, longitudinal study
Source: Transl Psychiatry. 2024 Feb 22;14:106. doi: 10.1038/s41398-024-02818-x (PMC10884408; doi:10.1038/s41398-024-02818-x)
Supplement: Supplementary file 1 — Supplemental Material [file 41398_2024_2818_MOESM1_ESM.pdf]

Neural Sensitivity following Stress Predicts Anhedonia Symptoms:

A 2-Year Multi-Wave, Longitudinal Study

Supplementary Material

Jaclyn S. Kirshenbaum[1,2], David Pagliaccio[1,2], Diego A. Pizzagalli[3,4], & Randy P. Auerbach[1,2,5]

[1]Department of Psychiatry, Columbia University, New York, New York

[2]Division of Child and Adolescent Psychiatry, New York State Psychiatric Institute, New York, New York

[3]Center for Depression, Anxiety and Stress Research, McLean Hospital, Belmont, Massachusetts

[4]Department of Psychiatry, Harvard Medical School, Boston, Massachusetts

[5]Division of Clinical Developmental Neuroscience, Sackler Institute, New York, NY

Anatomical Data Preprocessing

The T1-weighted (T1w) images were corrected for intensity non-uniformity (INU) with N4BiasFieldCorrection [1], distributed with ANTs 2.2.0 [2] (RRID:SCR_004757), and used as T1w-reference throughout the workflow. The T1w-reference was then skull-stripped with a Nipype implementation of the antsBrainExtraction.sh workflow (from ANTs), using OASIS30ANTs as target template. Brain tissue segmentation of cerebrospinal fluid (CSF), white-matter (WM) and gray-matter (GM) was performed on the brain-extracted T1w using fast (FSL 5.0.9, RRID:SCR_002823) [3]. Brain surfaces were reconstructed using recon-all (FreeSurfer 6.0.1, RRID:SCR_001847) [4], and the brain mask estimated previously was refined with a custom variation of the method to reconcile ANTs-derived and FreeSurfer-derived segmentations of the cortical gray-matter of Mindboggle (RRID:SCR_002438) [5]. Volume-based spatial normalization to one standard space (MNI152NLin2009cAsym) was performed through nonlinear registration with antsRegistration (ANTs 2.2.0), using brain-extracted versions of both T1w reference and the T1w template. The following template was selected for spatial normalization: *ICBM 152 Nonlinear Asymmetrical template version 2009c* [6] [RRID:SCR_008796; TemplateFlow ID: MNI152NLin2009cAsym].

Functional Data Preprocessing

First, a reference volume and its skull-stripped version were generated using a custom methodology of fMRIPrep. A B0-nonuniformity map (or fieldmap) was estimated based on a phase-difference map calculated with a dual-echo GRE (gradient-recall echo) sequence, processed with a custom workflow of SDCFlows inspired by the epidewarp.fsl script and further improvements in HCP Pipelines [7]. The fieldmap was then co-registered to the target EPI (echo-planar imaging) reference run and converted to a displacements field map (amenable to registration tools such as ANTs) with FSL's fugue and other *SDCflows* tools. Based on the estimated susceptibility distortion, a corrected EPI (echo-planar imaging) reference was calculated for a more accurate co-registration with the anatomical reference. The BOLD

reference was then co-registered to the T1w reference using bbregister (FreeSurfer) which implements boundary-based registration [8]. Co-registration was configured with six degrees of freedom. Head-motion parameters with respect to the BOLD reference (transformation matrices, and six corresponding rotation and translation parameters) are estimated before any spatiotemporal filtering using mcflirt (FSL 5.0.9) [9]. BOLD runs were slice-time corrected using 3dTshift from AFNI 20160207 [10] (RRID:SCR_005927).

The BOLD time-series (including slice-timing correction when applied) were resampled onto their original, native space by applying a single, composite transform to correct for head-motion and susceptibility distortions. These resampled BOLD time-series will be referred to as preprocessed BOLD in original space, or just preprocessed BOLD. The BOLD time-series were resampled into standard space, generating a preprocessed BOLD run in ['MNI152NLin2009cAsym'] space. A reference volume and its skull-stripped version were then generated using a custom methodology of fMRIPrep.

Several confounding time-series were calculated based on the *preprocessed BOLD*: framewise displacement (FD), DVARS and three region-wise global signals. FD and DVARS are calculated for each functional run, both using their implementations in Nipype (following the definitions by Power et al., 2014 [11]. The three global signals are extracted within the CSF, the WM, and the whole-brain masks. Additionally, a set of physiological regressors were extracted to allow for component-based noise correction (CompCor, [12]) Principal components are estimated after high-pass filtering the *preprocessed BOLD* time-series (using a discrete cosine filter with 128s cut-off) for the two CompCor variants: temporal (tCompCor) and anatomical (aCompCor). tCompCor components are then calculated from the top 5% variable voxels within a mask covering the subcortical regions. This subcortical mask is obtained by heavily eroding the brain mask, which ensures it does not include cortical GM regions. For aCompCor, components are calculated within the intersection of the aforementioned mask and the union of CSF and WM masks calculated in T1w space, after their projection to the native space of each

functional run (using the inverse BOLD-to-T1w transformation). Components are also calculated separately within the WM and CSF masks. For each CompCor decomposition, the *k* components with the largest singular values are retained, such that the retained components' time series are sufficient to explain 50 percent of variance across the nuisance mask (CSF, WM, combined, or temporal). The remaining components are dropped from consideration. The 6-parameter head-motion estimates calculated in the correction step were placed within the corresponding confounds file. The confound time series derived from head motion estimates and global signals were expanded with the inclusion of temporal derivatives and quadratic terms for each [13], resulting in 32 motion confounds. Frames that exceeded a threshold of 0.5 mm FD or 1.5 standardised DVARS were annotated as motion outliers. All resamplings were performed with a single interpolation step by composing all the pertinent transformations (i.e., head-motion transform matrices, susceptibility distortion correction when available, and co-registrations to anatomical and output spaces). Gridded (volumetric) resamplings were performed using antsApplyTransforms (ANTs), configured with Lanczos interpolation to minimize the smoothing effects of other kernels [14]. Non-gridded (surface) resamplings were performed using mri_vol2surf (FreeSurfer).

Supplementary Figure 1. Change in ROI activation pre- to post-stress

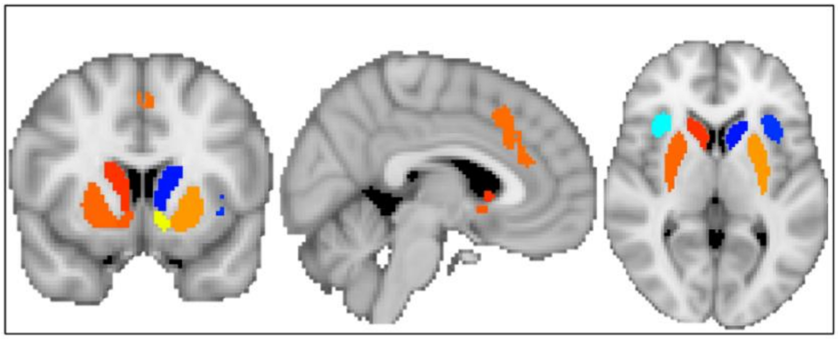

There were no outliers in residual scores, defined by values exceeding Q3 ± 3*IQR. On average, there was no evidence of a significant difference in ROI activation pre- to post-stress in the Win or Loss Conditions (see Supplementary Figures 2A, 3A); however, there is variation in individual change in activation pre- to post-stress in both conditions (see Supplementary Figures 2B, 3B).

Supplementary Figure 2. ROI activation patterns pre- and post-stress within the win condition

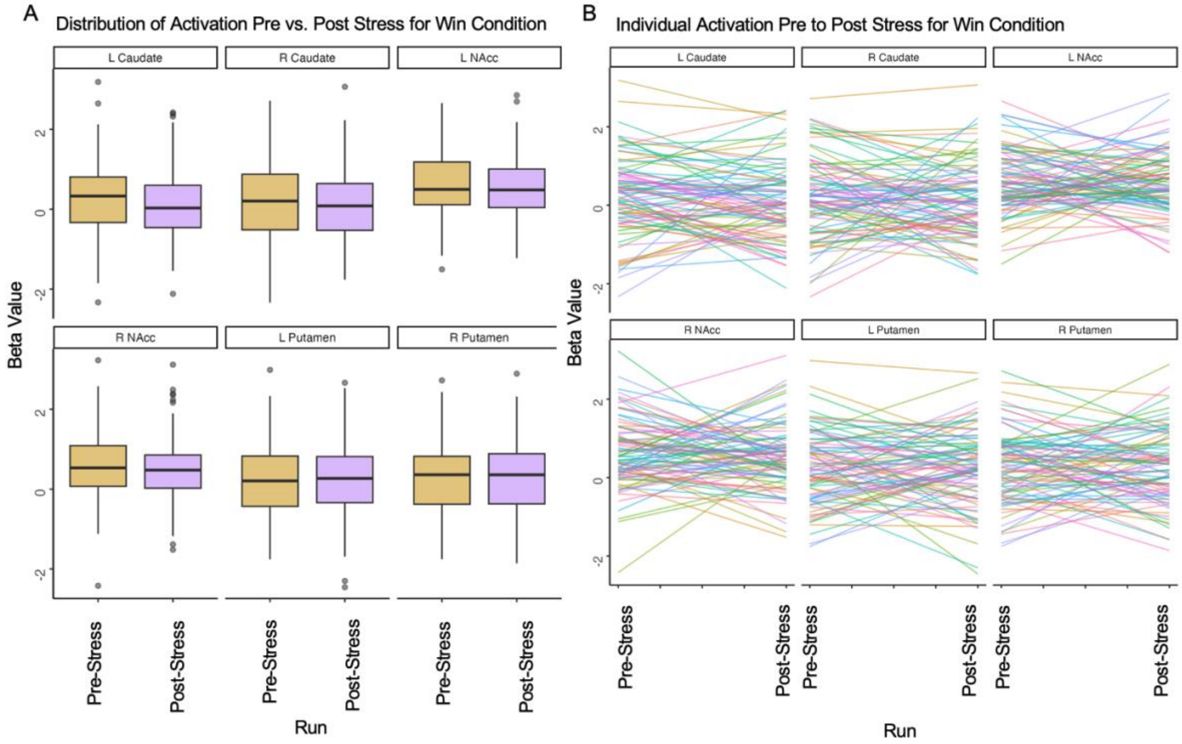

Note. R=Right; L=Left

Supplementary Figure 3. ROI activation patterns pre- and post-stress within the loss condition

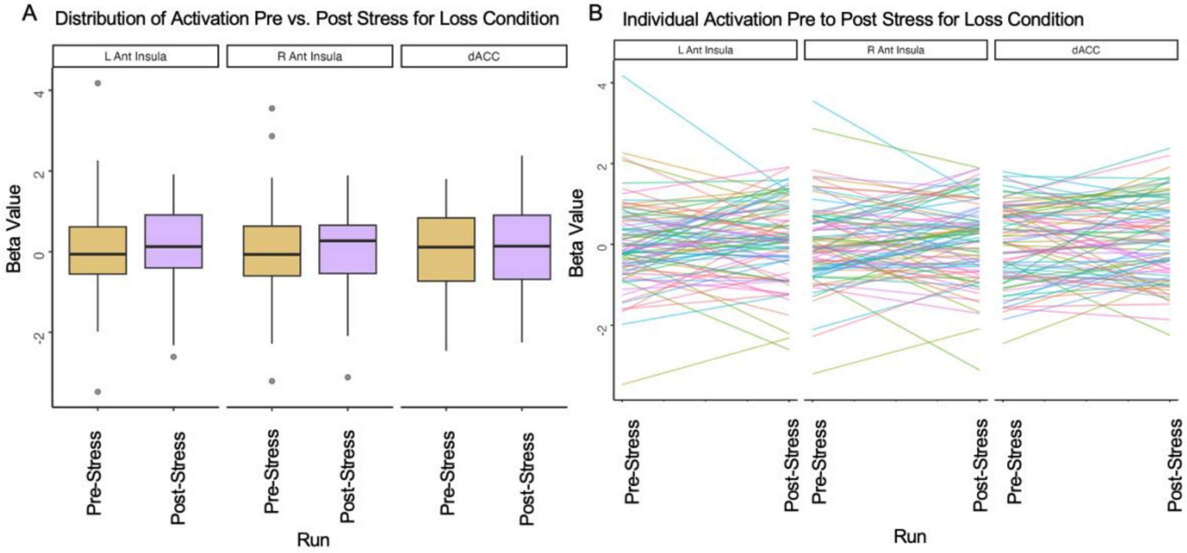

Note. R=Right; L=Left

Supplementary Figure 4. Affective ratings pre and post stress

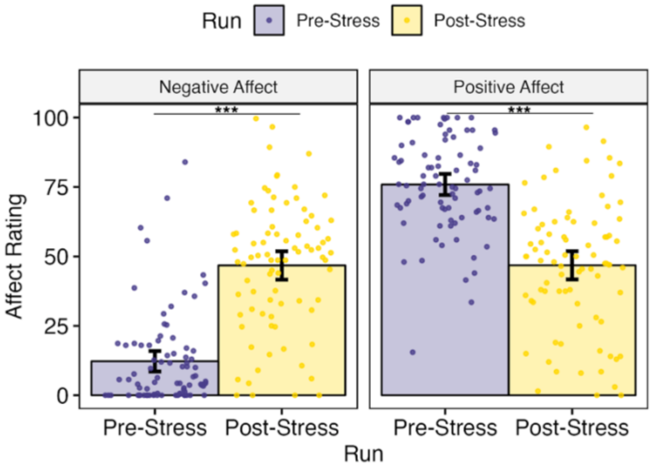

Note. Height of bars indicate mean affect rating, and bars denote 95% CI. ****p*<0.001.

Supplementary Table 1. Comparison of Participant Characteristics between Included and Excluded Participants

Variable	Sample		Group Difference
	Excluded (n=64)	Included (n=85)	
High Risk (%)	31	25	X2=0.49, p=0.484
Age (M, SD)	12.95, 0.84	12.99, 0.79	t=-0.27, p=0.790
Tanner Score (M, SD)	3.02, 0.73	3.04, 0.60	t=-0.18, p=0.855
Sex: Female (%)	66	62	X2=0.14, p=0.713
Ethnicity: Hispanic (%)	8	4	X2=0.73, p=0.393
Race (%)			
Asian	3	5	
American Indian	2	0	
Black	8	0	X2=13.88, p=0.008
Multiracial	19	8	
White	64	87	
Income (%)			
<10k	2	0	
10-25k	2	0	
25-50k	6	0	
50-75k	8	7	X2=10.06.97, p=0.122
75-100k	14	15	
100k+	58	67	
Unknown	5	11	
Anhedonia (M, SD)	22.00 (4.89)	22.01 (4.64)	t=-0.01, p=0.990
Depression (M, SD)	7.43 (6.06)	7.05 (6.51)	t=-0.32, p=0.753
Anxiety (M, SD)	38.03 (13.70)	35.75 (12.03)	t=0.87, p=0.386
Stress (M, SD)	20.03 (15.10)	20.04 (14.14)	t=-0.003, p=0.998

Note. Choices for biological sex were Male or Female. Within those excluded, some demographic information was not recorded (*n*=27) with unrecorded Tanner, SHAPS, MAFQ, MASC, ALEQ, *n*=1 with unrecorded Sex, *n*=3 with unrecorded Ethnicity and Race. Post-hoc comparisons for the significant group difference in Race are unreliable because there are rows with zero counts.

Supplementary Table 2. Comparison of Participant Characteristics between Included and Excluded Participants with Brain Data

Variable	Sample		Group Difference
	Excluded (n=24)	Included (n=85)	
High Risk (%)	21	25	χ^2=0.01, *p*=0.903
Age (M, SD)	12.63, 0.77	12.99, 0.79	*t*=-2.03, *p*=0.050
Tanner Score (M, SD)	2.99, 0.72	3.04, 0.60	*t*=-0.33, *p* =0.743
Sex: Female (%)	50	62	χ^2=0.73, *p*=0.393
Ethnicity: Hispanic (%)	8	4	χ^2=0.19, *p*=0.659
Race (%)			
Asian	0	5	
Black	0	0	
Multiracial	12	8	χ^2=1.50, *p*=0.472
White	88	87	
Income (%)			
50-75k	8	7	
75-100k	25	15	
100k+	58	67	χ^2=1.37, *p*=0.714
Unknown	8	11	
Anhedonia (M, SD)	22.04 (5.50)	22.01 (4.64)	*t*=0.02, *p*=0.981
Depression (M, SD)	7 (6.06)	7.05 (6.51)	*t*=-0.03, *p*=0.974
Anxiety (M, SD)	36 (12.78)	35.75 (12.03)	*t*=0.08, *p*=0.933
Stress (M, SD)	19.63 (12.86)	20.04 (14.14)	*t*=-0.13, *p*=0.893

Note. Choices for biological sex were Male or Female.

Supplementary Table 3. Overview of Clinical Measures Across Assessments

**Month after baseline**	**Measure**		
	SHAPS M (SD)	MFQ M (SD)	MASC M (SD)
0	22.01 (4.64)	6.96 (6.21)	38.38 (11.75)
1	20.69 (4.08)	5.76 (5.27)	36.78 (12.54)
3	20.19 (4.53)	5.86 (5.39)	37.6 (13.76)
6	20.35 (4.35)	5.47 (5.58)	36.06 (12.81)
9	20.82 (4.67)	5.76 (5.83)	37.53 (12.41)
12	20.27 (4.54)	5.68 (4.94)	35.97 (12.37)
15	19.88 (5.12)	6.27 (6.38)	38.14 (14.15)
18	20.12 (5.07)	5.98 (6.02)	36.73 (14.17)
21	21.13 (4.53)	6.57 (6.13)	37.59 (14.17)
24	21.16 (4.83)	5.99 (6.13)	36.63 (14.28)

Note. Month 0 = baseline; SHAPS=Snaith-Hamilton Pleasure Scale; MFQ=Mood and Feelings Questionnaire; MASC=Multidimensional Anxiety Scale for Children

Supplementary Table 4. Sensitivity Analyses Removing Participants without Full ROI Coverage

	Anhedonia Severity								
Predictors	*β*	*95% CI*	*p*	*β*	*95% CI*	*p*	*β*	*95% CI*	*p*
Intercept	0.12	-0.14 – 0.38	0.404	0.14	-0.11 – 0.40	0.294	0.14	-0.14 – 0.40	0.326
Age	-0.22	-0.36 – -0.06	**0.006**	-0.19	-0.33 – -0.04	**0.016**	-0.22	-0.36 – -0.06	**0.01**
Risk Group [High]	0.7	0.32 – 1.09	**<0.001**	0.67	0.29 – 1.06	**<0.001**	0.69	0.31 – 1.07	**<0.001**
Sex [F]	-0.41	-0.75 – -0.07	**0.02**	-0.44	-0.77 – -0.11	**0.016**	-0.44	-0.77 – -0.10	**0.018**
Between-person Stress	0.15	0.01 – 0.31	**0.042**	0.03	-0.13 – 0.19	0.708	0.14	-0.01 – 0.30	0.084
Within-person Stress	0.09	0.04 – 0.14	**<0.001**	0.02	-0.03 – 0.08	0.426	0.08	0.03 – 0.14	**0.002**
R NAcc	0.07	-0.09 – 0.22	0.408	0.06	-0.09 – 0.22	0.414	0.07	-0.09 – 0.22	0.382
Within-person Stress*R NAcc	-0.07	-0.13 – -0.01	**0.016**	-0.07	-0.13 – -0.02	**0.01**	-0.07	-0.12 – -0.01	**0.018**
Depression				0.24	0.15 – 0.33	**<0.001**			
Anxiety							0.04	-0.04 – 0.15	0.364
**Random Effects**									
σ^2	0.34			0.32			0.34		
τ_{00}	0.47_{ID}			0.44_{ID}			0.46_{ID}		
τ_{11}	$0.05_{ID.visit}$			$0.05_{ID.visit}$			$0.05_{ID.visit}$		
ρ_{01}	0.17_{ID}			0.08_{ID}			0.17_{ID}		
ICC	0.58			0.58			0.58		
N	82_{ID}			82_{ID}			82_{ID}		
Observations	629			629			629		
Marginal R^2 / Conditional R^2	0.202 / 0.666			0.244 / 0.683			0.206 / 0.665		

Note. σ2=residual variance; τ00=between-subject variance; τ11=random-slope variance; ρ01=random-slope-intercept-correlation; ICC=intraclass-correlation coefficient.

Supplementary Table 5. Anhedonia Model: Null Win-related Brain x Stress Results

	Anhedonia Severity									
Predictors	*β*	*95% CI*	*β*	*95% CI*	*β*	*95% CI*	*β*	*95% CI*	*β*	*95% CI*
Intercept	0.11	-0.15 – 0.36	0.18	-0.08 – 0.42	0.18	-0.07 – 0.42	0.12	-0.14 – 0.37	0.17	-0.08 – 0.43
Age	**-0.23**	**-0.38 – -0.08**	**-0.25**	**-0.39 – -0.09**	**-0.24**	**-0.38 – -0.10**	**-0.23**	**-0.38 – -0.07**	**-0.24**	**-0.38 – -0.08**
Risk Group [High]	**0.67**	**0.33 – 1.02**	**0.63**	**0.30 – 0.96**	**0.6**	**0.28 – 0.94**	**0.66**	**0.33 – 0.99**	**0.64**	**0.31 – 0.97**
Sex [F]	**-0.41**	**-0.72 – -0.09**	**-0.51**	**-0.81 – -0.19**	**-0.51**	**-0.80 – -0.20**	**-0.42**	**-0.72 – -0.11**	**-0.5**	**-0.79 – -0.19**
Between-person Stress	**0.16**	**0.01 – 0.31**	**0.19**	**0.04 – 0.34**	**0.19**	**0.05 – 0.33**	**0.16**	**0.01 – 0.31**	**0.17**	**0.03 – 0.32**
Within-person Stress	**0.09**	**0.04 – 0.14**	**0.09**	**0.04 – 0.14**	**0.09**	**0.04 – 0.14**	**0.09**	**0.04 – 0.14**	**0.09**	**0.04 – 0.14**
L NAcc	-0.01	-0.16 – 0.14								
Within-Person Stress* L NAcc	-0.03	-0.09 – 0.02								
L Caudate			**0.22**	**0.06 – 0.38**						
Within-Person Stress* L Caudate			0	-0.06 – 0.06						
R Caudate					**0.26**	**0.11 – 0.40**				
Within-Person Stress* R Caudate					0	-0.05 – 0.06				
L Putamen							0.13	-0.04 – 0.29		
Within-Person Stress* L Putamen							-0.01	-0.07 – 0.05		
R Putamen									**0.2**	**0.06 – 0.35**
Within-Person Stress* R Putamen									0	-0.05 – 0.06
**Random Effects**										
σ^2	0.33		0.33		0.33		0.33		0.33	
τ_{00}	0.46 $_{ID}$		0.41 $_{ID}$		0.40 $_{ID}$		0.44 $_{ID}$		0.42 $_{ID}$	
τ_{11}	0.05 $_{ID.visit}$		0.05 $_{ID.visit}$		0.05 $_{ID.visit}$		0.05 $_{ID.visit}$		0.05 $_{ID.visit}$	
ρ_{01}	0.12 $_{ID}$		0.10 $_{ID}$		0.13 $_{ID}$		0.13 $_{ID}$		0.14 $_{ID}$	
Sex [F]	0.58		0.56		0.55		0.57		0.56	
N	85 $_{ID}$		85 $_{ID}$		85 $_{ID}$		85 $_{ID}$		85 $_{ID}$	
Observations	653		653		653		653		653	

Marginal R^2 / Conditional R^2	0.204 / 0.665	0.244 / 0.664	0.260 / 0.663	0.219 / 0.664	0.239 / 0.662

Note. Bolded values indicate significant results, *p*<0.05. σ2=residual variance; τ00=between-subject variance; τ11=random-slope variance; ρ01=random-slope-intercept-correlation; ICC=intraclass-correlation coefficient.

Supplementary Table 6. Anhedonia Model: Null Loss-related Brain x Stress Results

	Anhedonia Severity					
Predictors	*β*	*95% CI*	*β*	*95% CI*	*β*	*95% CI*
Intercept	0.16	-0.11 – 0.42	0.11	-0.14 – 0.36	0.11	-0.15 – 0.37
Age	**-0.23**	**-0.37 – -0.08**	**-0.24**	**-0.38 – -0.08**	**-0.23**	**-0.38 – -0.07**
Risk Group [High]	**0.69**	**0.36 – 1.02**	**0.68**	**0.35 – 1.02**	**0.7**	**0.35 – 1.04**
Sex [F]	**-0.49**	**-0.81 – -0.17**	**-0.41**	**-0.72 – -0.11**	**-0.41**	**-0.72 – -0.11**
Between-person Stress	0.15	-0.00 – 0.29	0.15	0.00 – 0.30	**0.15**	**0.01 – 0.31**
Within-person Stress	**0.09**	**0.04 – 0.14**	**0.09**	**0.04 – 0.14**	**0.09**	**0.04 – 0.14**
dACC	0.19	-0.00 – 0.38				
Within-Person Stress* dACC	0.01	-0.06 – 0.07				
L Anterior Insula			0.16	-0.01 – 0.33		
Within-Person Stress* L Anterior Insula			-0.02	-0.08 – 0.04		
R Anterior Insula					0.11	-0.07 – 0.28
Within-Person Stress* R Anterior Insula					-0.01	-0.07 – 0.06
**Random Effects**						
σ^2	0.33		0.33		0.33	
τ_{00}	0.43 $_{ID}$		0.43 $_{ID}$		0.45 $_{ID}$	
τ_{11}	0.05 $_{ID.visit}$		0.05 $_{ID.visit}$		0.05 $_{ID.visit}$	
ρ_{01}	0.12 $_{ID}$		0.12 $_{ID}$		0.12 $_{ID}$	
ICC	0.57		0.57		0.57	
N	85 $_{ID}$		85 $_{ID}$		85 $_{ID}$	
Observations	653		653		653	
Marginal R^2 / Conditional R^2	0.223 / 0.663		0.226 / 0.665		0.211 / 0.664	

Note. Bolded values indicate significant results, p<0.05. σ2=residual variance; τ00=between-subject variance; τ11=random-slope variance; ρ01=random-slope-intercept-correlation; ICC=intraclass-correlation coefficient.

Supplementary Table 7. Sensitivity Analyses Removing Participants without Full ROI Coverage

	Depression Severity								
Predictors	*β*	*95% CI*	*p*	*β*	*95% CI*	*p*	*β*	*95% CI*	*p*
Intercept	-0.01	-0.14 – 0.12	0.914	0	-0.12 – 0.10	0.952	0	-0.13 – 0.12	0.946
Age	-0.19	-0.35 – -0.05	**0.004**	-0.18	-0.31 – -0.06	**<0.001**	-0.16	-0.31 – -0.01	**0.028**
Tanner	0.17	0.02 – 0.33	**0.03**	0.2	0.07 – 0.34	**<0.001**	0.18	0.02 – 0.33	**0.018**
Between-person Stress	0.59	0.48 – 0.71	**<0.001**	0.42	0.33 – 0.53	**<0.001**	0.56	0.45 – 0.68	**<0.001**
Within-person Stress	0.28	0.24 – 0.32	**<0.001**	0.22	0.18 – 0.26	**<0.001**	0.26	0.22 – 0.30	**<0.001**
R Putamen	0.03	-0.10 – 0.16	0.694	0	-0.11 – 0.11	0.992	0.01	-0.12 – 0.13	0.936
Within-person Stress*R Putamen	0.08	0.04 – 0.13	**<0.001**	0.08	0.04 – 0.13	**<0.001**	0.08	0.04 – 0.13	**<0.001**
Anxiety				0.39	0.32 – 0.46	**<0.001**			
Anhedonia							0.15	0.08 – 0.21	**<0.001**
**Random Effects**									
σ^2	0.22			0.19			0.21		
τ_{00}	0.30 $_{ID}$			0.22 $_{ID}$			0.29 $_{ID}$		
τ_{11}	0.05 $_{ID.visit}$			0.04 $_{ID.visit}$			0.05 $_{ID.visit}$		
ρ_{01}	0.06 $_{ID}$			0.06 $_{ID}$			0.03 $_{ID}$		
ICC	0.58			0.54			0.58		
N	84 $_{ID}$			84 $_{ID}$			84 $_{ID}$		
Observations	648			648			646		
Marginal R^2 / Conditional R^2	0.463 / 0.774			0.586 / 0.808			0.485 / 0.781		

Note. σ2=residual variance; τ00=between-subject variance; τ11=random-slope variance; ρ01=random-slope-intercept-correlation; ICC=intraclass-correlation coefficient.

Supplementary Table 8. Depression Model: Null Win-related Brain x Stress Results

	Depression Severity			
Predictors	*β*	*95% CI*	*β*	*95% CI*
Intercept	-0.02	-0.15 – 0.10	-0.02	-0.15 – 0.10
Age	**-0.21**	**-0.35 – -0.06**	**-0.21**	**-0.35 – -0.06**
Tanner	**0.17**	**0.01 – 0.31**	**0.17**	**0.01 – 0.32**
Between-person Stress	**0.56**	**0.43 – 0.69**	**0.55**	**0.43 – 0.68**
Within-person Stress	**0.28**	**0.24 – 0.33**	**0.28**	**0.24 – 0.33**
L NAcc	0.01	-0.11 – 0.14		
Within-Person Stress* L NAcc	0	-0.05 – 0.05		
R NAcc			0	-0.13 – 0.13
Within-Person Stress* R NAcc			0.02	-0.02 – 0.07
**Random Effects**				
σ^2	0.24		0.24	
τ_{00}	0.31 $_{ID}$		0.31 $_{ID}$	
τ_{11}	0.06 $_{ID.visit}$		0.06 $_{ID.visit}$	
ρ_{01}	0.16 $_{ID}$		0.16 $_{ID}$	
ICC	0.56		0.56	
N	85 $_{ID}$		85 $_{ID}$	
Observations	655		655	
Marginal R^2 / Conditional R^2	0.432 / 0.749		0.432 / 0.750	

Note. Bolded values indicate significant results, $p<0.05$. σ2=residual variance; τ00=between-subject variance; τ11=random-slope variance; ρ01=random-slope-intercept-correlation; ICC=intraclass-correlation coefficient.

Supplementary Table 9. Depression Model: Null Loss-related Brain x Stress Results

	Depression Severity					
Predictors	*β*	*95% CI*	*β*	*95% CI*	*β*	*95% CI*
Intercept	-0.02	-0.15 – 0.10	-0.02	-0.15 – 0.10	-0.02	-0.15 – 0.10
Age	**-0.2**	**-0.35 – -0.06**	**-0.21**	**-0.35 – -0.06**	**-0.21**	**-0.35 – -0.06**
Tanner	0.16	0.00 – 0.31	**0.17**	**0.01 – 0.31**	**0.17**	**0.01 – 0.31**
Between-person Stress	**0.55**	**0.43 – 0.68**	**0.56**	**0.43 – 0.68**	**0.56**	**0.43 – 0.68**
Within-person Stress	**0.28**	**0.23 – 0.33**	**0.28**	**0.24 – 0.33**	**0.28**	**0.24 – 0.33**
dACC	0.03	-0.13 – 0.19				
Within-Person Stress* dACC	0.02	-0.03 – 0.07				
L Anterior Insula			-0.02	-0.17 – 0.13		
Within-Person Stress* L Anterior Insula			0.02	-0.04 – 0.07		
R Anterior Insula					-0.03	-0.17 – 0.12
Within-Person Stress* R Anterior Insula					0.01	-0.04 – 0.07
**Random Effects**						
σ^2	0.24		0.24		0.24	
τ_{00}	0.31 $_{ID}$		0.31 $_{ID}$		0.31 $_{ID}$	
τ_{11}	0.06 $_{ID.visit}$		0.06 $_{ID.visit}$		0.06 $_{ID.visit}$	
ρ_{01}	0.16 $_{ID}$		0.17 $_{ID}$		0.17 $_{ID}$	
ICC	0.56		0.56		0.56	
N	85 $_{ID}$		85 $_{ID}$		85 $_{ID}$	
Observations	655		655		655	
Marginal R^2 / Conditional R^2	0.432 / 0.750		0.431 / 0.750		0.432 / 0.749	

Note. Bolded values indicate significant results, $p<0.05$. σ2=residual variance; τ00=between-subject variance; τ11=random-slope variance; ρ01=random-slope-intercept-correlation; ICC=intraclass-correlation coefficient.

Supplementary Table 10. Anxiety Model: Null Win-related Brain x Stress Results

	Anxiety Severity											
Predictors	*β*	*95% CI*	*β*	*95% CI*	*β*	*95% CI*	*β*	*95% CI*	*β*	*95% CI*	*β*	*95% CI*
Intercept	**-0.34**	**-0.60 – -0.08**	**-0.34**	**-0.60 – -0.08**	**-0.34**	**-0.61 – -0.07**	**-0.33**	**-0.60 – -0.07**	**-0.34**	**-0.59 – -0.08**	**-0.35**	**-0.61 – -0.08**
Sex [F]	**0.55**	**0.24 – 0.87**	**0.55**	**0.24 – 0.87**	**0.55**	**0.22 – 0.88**	**0.54**	**0.21 – 0.88**	**0.55**	**0.24 – 0.87**	**0.56**	**0.25 – 0.89**
Between-person Stress	**0.35**	**0.19 – 0.50**	**0.35**	**0.20 – 0.51**	**0.35**	**0.20 – 0.51**	**0.36**	**0.20 – 0.52**	**0.35**	**0.20 – 0.51**	**0.35**	**0.20 – 0.51**
Within-person Stress	**0.15**	**0.10 – 0.18**	**0.15**	**0.10 – 0.19**	**0.15**	**0.11 – 0.19**	**0.15**	**0.11 – 0.19**	**0.15**	**0.11 – 0.19**	**0.15**	**0.11 – 0.19**
L NAcc	-0.05	-0.21 – 0.11										
Within-Person Stress* L NAcc	-0.05	-0.09 – -0.00										
R NAcc			-0.08	-0.24 – 0.09								
Within-Person Stress* R NAcc			-0.02	-0.06 – 0.02								
L Caudate					0.01	-0.16 – 0.18						
Within-Person Stress* L Caudate					0.02	-0.03 – 0.07						
R Caudate							0.02	-0.13 – 0.19				
Within-Person Stress* R Caudate							0.02	-0.03 – 0.06				

L Putamen									-0.04	-0.22 – 0.12		
Within-Person Stress* L Putamen									0	-0.05 – 0.04		
R Putamen											-0.04	-0.22 – 0.12
Within-Person Stress* R Putamen											0	-0.05 – 0.04
**Random Effects**												
σ^2	0.21		0.21		0.21		0.21		0.21		0.21	
τ_{00}	0.56 $_{ID}$		0.55 $_{ID}$		0.55 $_{ID}$		0.55 $_{ID}$		0.55 $_{ID}$		0.55 $_{ID}$	
τ_{11}	0.04 $_{ID.visit}$		0.04 $_{ID.visit}$		0.04 $_{ID.visit}$		0.04 $_{ID.visit}$		0.04 $_{ID.visit}$		0.04 $_{ID.visit}$	
ρ_{01}	0.37 $_{ID}$		0.35 $_{ID}$		0.33 $_{ID}$		0.33 $_{ID}$		0.34 $_{ID}$		0.35 $_{ID}$	
ICC	0.73		0.73		0.73		0.73		0.73		0.73	
N	85 $_{ID}$		85 $_{ID}$		85 $_{ID}$		85 $_{ID}$		85 $_{ID}$		85 $_{ID}$	
Observations	655		655		655		655		655		655	
Marginal R^2 / Conditional R^2	0.234 / 0.793		0.242 / 0.793		0.237 / 0.792		0.237 / 0.792		0.233 / 0.791		0.233 / 0.792	

Note. Bolded values indicate significant results, $p<0.05$. σ2=residual variance; τ00=between-subject variance; τ11=random-slope variance; ρ01=random-slope-intercept-correlation; ICC=intraclass-correlation coefficient.

Supplementary Table 11. Anxiety Model: Null Loss-related Brain x Stress Results

	Anxiety Severity	
Predictors	*β*	*95% CI*
Intercept	**-0.34**	**-0.60 – -0.08**
Sex [F]	**0.55**	**0.24 – 0.87**
Between-person Stress	**0.35**	**0.20 – 0.51**
Within-person Stress	**0.15**	**0.11 – 0.19**
L Anterior Insula	-0.01	-0.20 – 0.18
Within-Person Stress* L Anterior Insula	-0.03	-0.08 – 0.02
**Random Effects**		
σ^2	0.21	
$\tau_{00\ ID}$	0.55	
$\tau_{11\ ID.visit}$	0.04	
$\rho_{01\ ID}$	0.33	
ICC	0.73	
N_{ID}	85	
Observations	655	
Marginal R^2 / Conditional R^2	0.237 / 0.792	

Note. Bolded values indicate significant results, $p<0.05$. σ2=residual variance; τ00=between-subject variance; τ11=random-slope variance; ρ01=random-slope-intercept-correlation; ICC=intraclass-correlation coefficient.

References

1. Tustison NJ, Avants BB, Cook PA, Zheng Y, Egan A, Yushkevich PA, et al. N4ITK: Improved N3 Bias Correction. IEEE Trans Med Imaging. 2010;29:1310–1320.
2. Avants BB, Tustison N, Johnson H. Advanced Normalization Tools (ANTS). 2009. 2009.
3. Zhang Y, Brady M, Smith S. Segmentation of brain MR images through a hidden Markov random field model and the expectation-maximization algorithm. IEEE Trans Med Imaging. 2001;20:45–57.
4. Dale AM, Fischl B, Sereno MI. Cortical Surface-Based Analysis: I. Segmentation and Surface Reconstruction. NeuroImage. 1999;9:179–194.
5. Klein A, Ghosh SS, Bao FS, Giard J, Häme Y, Stavsky E, et al. Mindboggling morphometry of human brains. PLOS Comput Biol. 2017;13:e1005350.
6. Fonov V, Evans A, McKinstry R, Almli C, Collins D. Unbiased nonlinear average age-appropriate brain templates from birth to adulthood. NeuroImage. 2009;47:S102.
7. Glasser MF, Sotiropoulos SN, Wilson JA, Coalson TS, Fischl B, Andersson JL, et al. The minimal preprocessing pipelines for the Human Connectome Project. NeuroImage. 2013;80:105–124.
8. Greve DN, Fischl B. Accurate and robust brain image alignment using boundary-based registration. NeuroImage. 2009;48:63–72.
9. Jenkinson M, Bannister P, Brady M, Smith S. Improved optimization for the robust and accurate linear registration and motion correction of brain images. NeuroImage. 2002;17:825–841.
10. Cox RW, Hyde JS. Software tools for analysis and visualization of fMRI data. NMR Biomed. 1997;10:171–178.
11. Power JD, Mitra A, Laumann TO, Snyder AZ, Schlaggar BL, Petersen SE. Methods to detect, characterize, and remove motion artifact in resting state fMRI. NeuroImage. 2014;84.
12. Behzadi Y, Restom K, Liau J, Liu TT. A component based noise correction method (CompCor) for BOLD and perfusion based fMRI. NeuroImage. 2007;37:90–101.
13. Satterthwaite TD, Elliott MA, Gerraty RT, Ruparel K, Loughead J, Calkins ME, et al. An improved framework for confound regression and filtering for control of motion artifact in the preprocessing of resting-state functional connectivity data. NeuroImage. 2013;64:240–256.
14. Lanczos C. Evaluation of Noisy Data. J Soc Ind Appl Math Ser B Numer Anal. 1964;1:76–85.
